# Glycogenic Hepatopathy Masquerading as Acute Pancreatitis

**DOI:** 10.7759/cureus.13397

**Published:** 2021-02-17

**Authors:** Steven H Adams, Michelle Bernshteyn, Umair Masood, James Corines, Divey Manocha

**Affiliations:** 1 Pathology, State University of New York Upstate Medical University, Syracuse, USA; 2 Internal Medicine, State University of New York Upstate Medical University, Syracuse, USA; 3 Gastroenterology, State University of New York Upstate Medical University, Syracuse, USA

**Keywords:** pancreatitis, hepatomegaly, glycogenic hepatopathy, mauriac syndrome, type 1 diabetes mellitus (t1dm)

## Abstract

Glycogenic hepatopathy (GH), defined histologically by hepatocytic glycogen accumulation without fatty change or fibrosis, is a benign reversible condition. It presents clinically as hepatomegaly with elevated liver enzymes in young diabetic (type 1) patients with poor glycemic control. We report a case of a 20-year-old female with a history of poorly controlled type 1 diabetes mellitus (T1DM) and prior pancreatitis who presented with sharp epigastric pain and hepatomegaly. She was found to have diabetic ketoacidosis with elevated lipase and amylase. Though at first her symptoms were erroneously attributed to pancreatitis, a liver biopsy showing glycogenated nuclei led to a diagnosis of GH.

## Introduction

GH, an uncommon complication of chronic poorly controlled T1DM in pediatric patients, is underrecognized amongst clinicians [1,2]. It is often confused with nonalcoholic fatty liver disease (NAFLD), which can present similarly. Unlike NAFLD, which may progress to fibrosis or cirrhosis, GH resolves with dysglycemia control. We present a case of GH that uniquely appeared clinically at first as acute pancreatitis. Imaging and ultimately biopsy clarified the diagnosis.

This article was previously presented as a meeting abstract at the 2020 American College of Gastroenterology Conference on October 27, 2020.

## Case presentation

A 20-year-old female with a past medical history of poorly controlled type 1 diabetes mellitus (T1DM) and prior episode of pancreatitis presented with sharp epigastric pain that worsened by eating. The patient was tachycardic to 115 beats per minute, and physical examination revealed tenderness in the right upper quadrant and epigastric region with negative Murphy’s sign. Laboratory workup was significant for a white blood cell count of 12,900/μL, lipase of 353 U/L (with an upper limit of normal of 60 U/L), amylase of 264 U/L (with an upper limit of normal of 103 U/L), beta-hydroxybutyrate of 3.02 mmol/L, hemoglobin A1c (HbA1c) of 13.5, alanine transaminase (ALT) of 34 U/L and aspartate transaminase (AST) of 34 U/L, alkaline phosphatase of 191, triglycerides of 162 mg/dL, and lactate of 5.4 mmol/L, which later increased to 8.2 mmol/L. Repeat hepatic panel demonstrated ALT of 24 U/L and AST of 51 U/L. Hepatitis panel, iron panel, antinuclear antibodies (ANA), anti-smooth muscle antibody, and ceruloplasmin were negative.

Computed tomography (CT) of the abdomen revealed hepatomegaly of approximately 24-25 cm craniocaudally, hepatic steatosis, trace pelvic ascites, and normal pancreas. Abdominal ultrasound showed an enlarged liver with diffuse increase in parenchymal echogenicity, suggestive of steatosis, without any focal lesions. Mesenteric vascular Doppler showed normal vasculature and flow.

**Figure 1:**
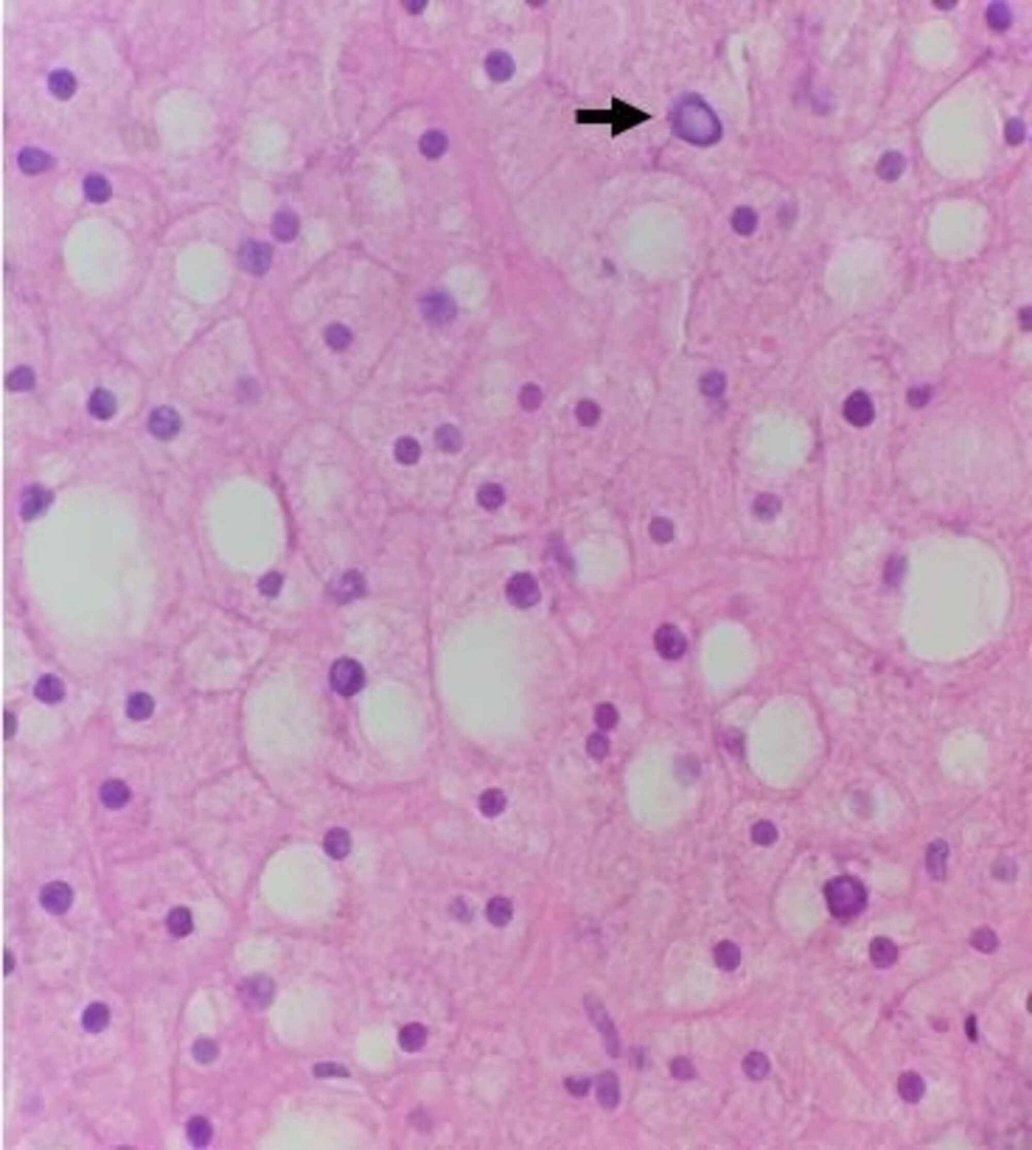
Hematoxylin and eosin (H&E), high magnification. Benign liver parenchyma with focal mild macrovascular steatosis can be seen. Many hepatocytes have increased swelling and cytoplasmic clearing. Occasional glycogenated nuclei are seen (as indicated by the black arrow).

Liver biopsy showed preserved parenchyma without fibrosis, focal mild macrovesicular steatosis, and occasional glycogenated nuclei (Figure 1).

**Figure 2:**
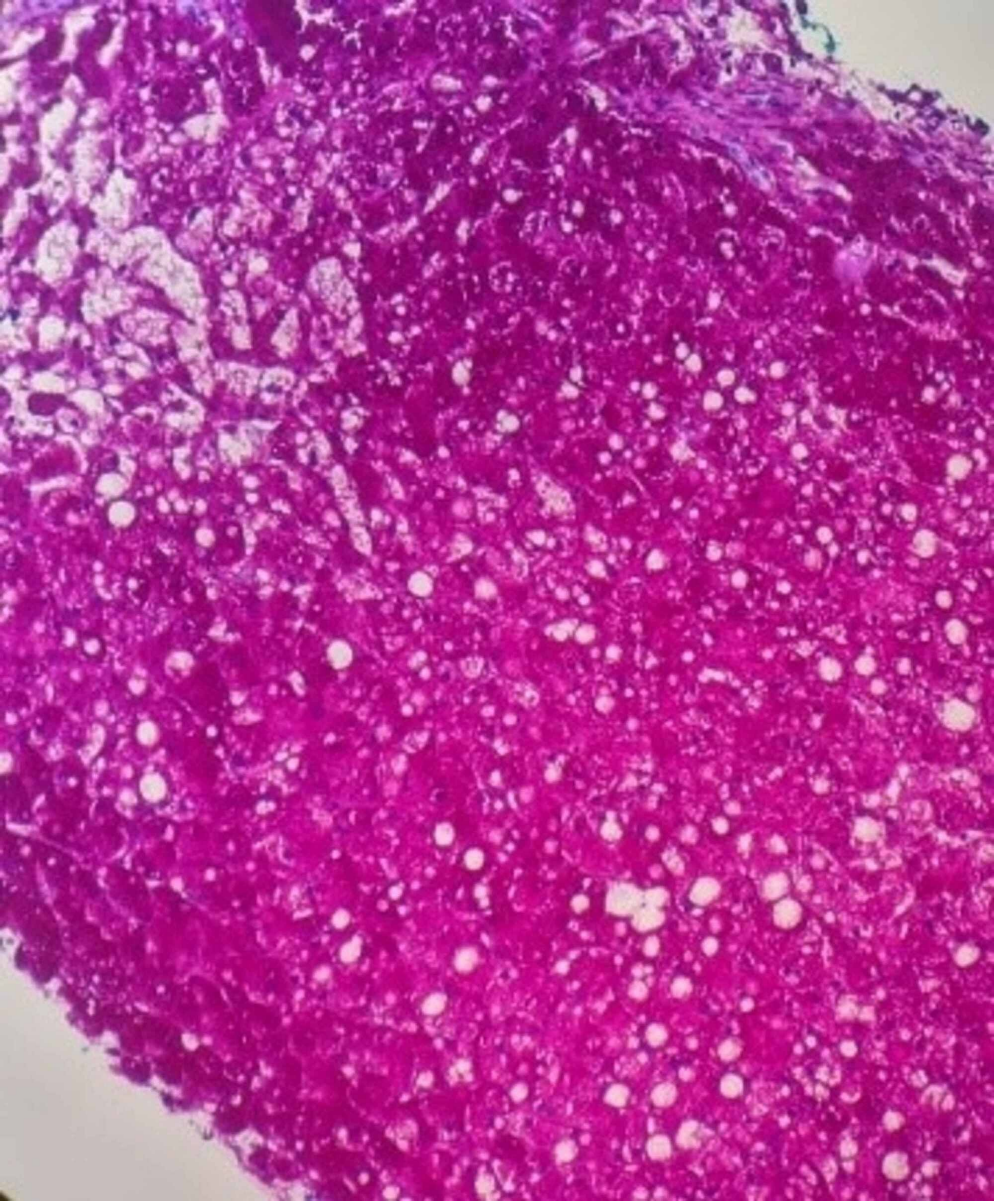
A PAS stain highlighting abundant glycogen in the hepatocytes, as evidenced by the magenta staining, but is not seen in the PASD stain (PAS stain after diastase pretreatment). PAS, Periodic acid-Schiff

The hepatocytes had increased swelling and cytoplasmic clearing, suggestive of glycogen deposition. Periodic acid-Schiff (PAS) stain revealed abundant hepatocyte glycogen deposits, which were not visible after diastase digestion (Figure 2).

The histological findings are consistent with a GH diagnosis.

Diabetic ketoacidosis resolved with intravenous, followed by subcutaneous, insulin, and the patient’s diet was slowly advanced. Lactic acidosis resolved with the administration of intravenous fluids. Furthermore, liver function tests improved prior to discharge. Endocrinology consultation was placed for adjustment of insulin regimen. Throughout hospitalization, the patient reported no further episodes of epigastric pain and was discharged home with a plan for close follow-up.

## Discussion

GH, which is characterized by tender hepatomegaly with elevated liver enzymes in the setting of diabetes mellitus, exists within the Mauriac Syndrome spectrum, but it lacks the syndrome’s additional cushingoid features and poor growth. GH almost always occurs with T1DM and has slight female predominance.

Common clinical features include symptoms of diabetic ketoacidosis, such as abdominal pain, nausea, vomiting, polyuria, and polydipsia. Also common are signs of hepatic pathology, including hepatomegaly, jaundice, pruritis, and ascites [1-4]. The pathobiology of GH may involve oscillation of glucose levels with poor insulin management leading to hepatocyte glucose trapping and glycogenosis [1]. Biochemical findings include elevated HbA1c, AST greater than ALT, and elevated alkaline phosphatase [1,2]. It is unusual to see ALT or AST levels lower than 100 U/L [1,3,4]. (Histological evidence suggests that elevations in liver enzymes are caused by leakage from hepatocyte membrane injury and not liver cell necrosis [2].) Increased amylase and lipase levels have been reported, as seen in our patient [3]. Diabetic ketoacidosis (DKA) and lactic acidosis are common findings [4]. Ascites has been rarely reported [1].

The differential diagnosis of hepatitis in a patient with T1DM includes hemochromatosis, Wilson’s disease, autoimmune hepatitis, and nonalcoholic fatty liver disease (NAFLD). Definitive diagnosis is by findings of swollen hepatocytes with glycogen accumulation on biopsy, with limited or absent fatty change, inflammation, necrosis, or fibrosis [1]. It is important not to confuse GH with NAFLD. A clinical history of T2DM and obesity in adults points to NAFLD, while a history of T1DM in a thin pediatric patient is suggestive of GH [2]. CT scan of NAFLD shows a hypodense liver, whereas in GH, a hyperdense (bright) liver is seen [2,5]. Histological findings of fibrosis and lobular and portal inflammation are seen in NAFLD rather than GH. Unlike NAFLD, which may progress to fibrosis or cirrhosis, GH resolves with dysglycemia control [6].

In addition to GH being an infrequently encountered and underrecognized entity, our patient’s prior history of pancreatitis, together with her presenting symptoms of sharp epigastric pain worsened by eating (the classical clinical presentation of pancreatitis) and elevated pancreatic enzymes with near-normal liver enzymes (AST, ALT), initially mislead the clinical team into assuming that the patient was experiencing another episode of pancreatitis. Lack of pancreatic abnormalities on CT lead to performing a liver biopsy, which finally revealed the true diagnosis.

## Conclusions

Our case highlights the importance of recognition of the GH entity when encountering diabetic patients presenting with hepatomegaly and abdominal pain. It further encourages glycemic control to manage T1DM and complications such as GH.

## References

[1] Torbenson M, Chen YY, Brunt E (2006). Glycogenic hepatopathy: an underrecognized hepatic complication of diabetes mellitus. Am J Surg Pathol.

[2] Sherigar JM, de Castro J, Yin YM, Guss D, Mohanty SR (2018). Glycogenic hepatopathy: a narrative review. World J Hepatol.

[3] Silva M, Marques M, Cardoso H (2016). Glycogenic hepatopathy in young adults: a case series. Rev Esp Enferm Dig.

[4] Mukewar S, Sharma A, Lackore KA (2017). Clinical, biochemical, and histopathology features of patients with glycogenic hepatopathy. Clin Gastroenterol Hepatol.

[5] Sweetser S, Kraichely RE (2010). The bright liver of glycogenic hepatopathy. Hepatology.

[6] Al Sarkhy AA, Zaidi ZA, Babiker AM (2017). Glycogenic hepatopathy, an underdiagnosed cause of relapsing hepatitis in uncontrolled type 1 diabetes mellitus. Saudi Med J.

